# A Supramolecular Approach to Enhance the Optoelectronic Properties of P3HT-b-PEG Block Copolymer for Organic Field-Effect Transistors

**DOI:** 10.1021/acsomega.4c05648

**Published:** 2024-09-03

**Authors:** Pallavi Kumari, Barbara Hajduk, Paweł Jarka, Henryk Bednarski, Henryk Janeczek, Mieczysław Łapkowski, Sylwia Waśkiewicz

**Affiliations:** † Centre of Polymer and Carbon Materials, Polish Academy of Sciences, 34 Marie Curie Skłodowska Str., Zabrze 41−819, Poland; ‡ Department of Engineering Materials and Biomaterials, Silesian University of Technology, 18a Konarskiego Str., Gliwice 41−100, Poland; § Department of Physical Chemistry and Technology of Polymers, Faculty of Chemistry, Silesian University of Technology, M. Strzody 9, Gliwice 44−100, Poland

## Abstract

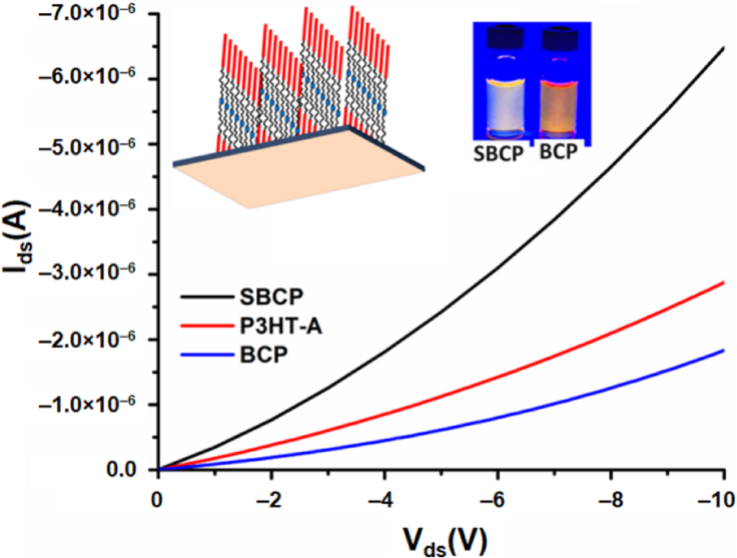

This study investigates a supramolecular approach to elucidate the interaction between an organic semiconducting molecule, specifically butyric acid-functionalized perylene diimide, and a block copolymer comprising poly-3-hexyl thiophene-b-polyethylene glycol. This interaction results in the formation of a precisely structured nanoarchitecture within the supramolecular block copolymer, driven by the ionic interplay between the block copolymer and small organic molecules. The optical properties of the synthesized supramolecular block copolymer were characterized by using ellipsometry. Additionally, further characterization employing atomic force microscopy, differential scanning calorimetry, and X-ray diffraction provided detailed insights into the crystallinity and morphology of the nanostructure. The characterization data showed that this approach significantly influenced the tuning of morphology, crystallinity, and optical and electronic properties of the resulting nanostructure. The demonstrated methodology holds considerable promise as a strategic tool for broadening the spectrum of attainable nanomorphologies in semiconducting polymers, particularly for applications in electronics or photovoltaics.

## Introduction

Organic semiconductors in thin-film transistors have gained significant attention and hold great promise due to their unique properties, versatility, and potential applications. Unlike traditional inorganic semiconductors, organic semiconductors offered several advantages in terms of flexibility, cost-effectiveness, and ease of processing.^1−3^ The incorporation of organic semiconductors into thin-film transistors presents a broad spectrum of potential applications, including wearable electronics, chemical sensors, and flexible display technologies.^4−6^ Conjugated polymers and organic small molecules have been extensively studied as semiconductors in order to achieve the requisite electrical characteristics.^7,8^ However, the efficiency of conjugated polymer-based devices is typically limited due to difficulties in managing their phase behavior and crystallinity. This limitation is primarily linked to the intrinsic chain rigidity of conjugated polymers, a factor that exerts a significant influence on both molecular packing and microstructure.^9^ Furthermore, organic semiconducting thin films are polycrystalline; therefore, mobility is mostly determined by the surface morphology, orientation, and size of the crystal grains.^10^ These aspects play a pivotal role in determining the performance of the device.^11,12^ Further to address this challenge, a distinctive strategy has been adopted through the synthesis of a conjugated rod-coil block copolymer. This approach intended to regulate the morphology and crystallization of the conjugated polymer, facilitating the development of flexible electronic devices.^13,14^

Furthermore, among conjugated polymers, poly(3-hexylthiophene) (P3HT) is recognized as a particularly promising material for organic field-effect transistors (OFETs) due to its elevated charge carrier mobility, compatibility with solution processing, and ease of synthesis.^15,16^ The synthesis of well-defined block copolymers (BCPs) incorporating P3HT blocks can be achieved through the controlled end functionalization of P3HT.^17^ Previous reports have described various P3HT-based block copolymers that include both polar and nonpolar blocks, such as P3HT-b-polyethylene glycol,^18^ P3HT-b-poly(*N*-isopropylamide),^19^ P3HT-b-polystyrene,^20^ P3HT-b- polymethylmethacrylate,^21^ and more. P3HT-based conjugated block copolymers with high P3HT contents (82–91%) have been shown to form well-ordered nanoarrays at the interface.^22,23^ Wang et al.^23^ designed a copolymer combining semiconducting P3HT with poly(butyl acrylate) to achieve high p-type mobility for use in stretchable electronics. This study demonstrated that incorporating the low glass transition temperature of poly(butyl acrylate) with P3HT enhances the semiconducting properties of P3HT in thin film and self-assembles into fibrillar-like nanostructures. This configuration maintains an edge-on orientation even at low P3HT compositions, which is crucial for effective charge transport in OFET. However, the insulating segment of the BCP limits the optical and electronic properties of the conjugated BCP.^24^

The self-assembly process is an effective method for enhancing the optical and electrical properties of BCP. This process involves the interaction between BCP and organic and inorganic semiconducting molecules through ionic interactions, which improve the optoelectronic properties of the conjugated BCP. Perylene diimide, an organic semiconducting molecule, has emerged as a promising material due to its excellent photophysical properties and thermal stability. It also demonstrating high electron affinity, favorable energy levels, and excellent electron-transporting properties, making it well-suited for application in organic electronics^25^ and contributing to enhanced stability.^26^

In OFETs, derivatives with a PDI core structure provide a rigid and planar conjugated system that leads to conjugation, facilitates close π–π stacking, and consequently enhances self-assembly properties, crystallinity, thermal stability, and optoelectronic properties.^27^ Previous studies have shown that incorporating pendent acceptor groups such as C_60_, isoindigo, and pyrene into P3HT-based rod-coil BCPs enhances their self-assembly behavior, optoelectronic properties, and flexibility.^28−31^ Despite existing research, there remains a gap in the study of P3HT-based supramolecular block copolymers, incorporating PDI derivatives, particularly evaluation of their optical properties, crystallinity, morphologies, and field-effect transistor characteristics. To address this gap, we aim to synthesize a supramolecular block copolymer (SBCP) composed of P3HT-alkyne (P3HT-A) with polyethylene glycol (PEG) using click chemistry. The PEG component, which has a lower glass transition temperature, was functionalized with both azido and amine moieties. The azide moiety facilitated the click chemistry reaction for synthesizing the BCP with P3HT-A, while the amine moiety contributed to the self-assembly process through ionic interactions with carboxyl-functionalized perylene diimide butyric acid (PDIBA). In the resulting BCP, P3HT constituted over 80% of the composition relative to PEG, ensuring that the PEG segment did not impede the electrical characteristics of P3HT. Furthermore, PDIBA actively participated in the self-assembly process with the block copolymer, thereby fine-tuning its properties. Therefore, our study primarily focused on investigating the optical properties, crystallinity, morphologies, and field-effect transistor characteristics of the synthesized SBCP.

## Materials and Methods

### Materials

Anhydrous toluene, tetrahydrofuran (THF) anhydrous, 2-bromo-5-iodo-3-hexyl thiophene, azide-amine-terminated poly(ethylene glycol) (azido-dPEG23-amine), isopropyl magnesium chloride (2.0 M in THF), ethynylmagnesium bromide (0.5 M in THF), [1,3-bis(diphenylphosphino)propane]dichloronickel(II)], copper bromide, *N*,*N*,*N*′,*N*′′,*N*′′-pentamethyldiethylenetriamine (PMDETA), 3,4,9,10-perylenetetracarboxylic dianhydride, 4-amino-*n* butyric acid, imidazole, and zinc acetate were purchased from Merck, Poland, and were used as received.

### Characterization

^1^H NMR spectra were recorded at 25 °C on a Bruker Avance II 600 MHz NMR spectrometer (Karlsruhe, Germany). Chemical shifts (δ) were reported in ppm. Infrared spectroscopy was conducted by using a PerkinElmer Spectrum Two spectrometer with a UATR module (Waltham, MA, USA). The molecular weight and polydispersity of the polymer were analyzed by using size-exclusion chromatography (Agilent HPLC 1260 Infinity system), employing THF as the eluent against polystyrene standards with a refractive index detector. The transmission spectra of thin films (in 240–2500 nm range) were taken with a spectroscopic ellipsometer (SENTECH SE850 spectrometer, Sentech, Krailling, Germany) in the transmission mode. The rest of the ellipsometric measurements were performed using the variable angle mode. The measurements were taken in the angular range of 50–70° at 5° intervals, and the dielectric functions were determined using Spectra Ray 3 software to operate the ellipsometer.

DSC Q2000 (TA Instruments, Newcastle, DE, USA) was used with aluminum sample pans to perform differential scanning calorimetry (DSC) measurements. The thermal characteristics of the samples were determined under a nitrogen atmosphere at a gas flow rate of 50 mL min^–1^. The instrument was calibrated with high-purity indium standards, and cooling and heating were conducted at a rate of 20 °C min^–1^. The X-ray diffraction (XRD) study was performed on polymer films using a D8 Advance diffractometer (Bruker, Karlsruhe, Germany) equipped with a Cu Kα cathode (λ = 1.54 Å) using the coupled Two-Theta/Theta mode. The measurement was taken at a rate of 1.2 °C min^–1^ with an angular step of 0.02° ranging from 2° to 60° for 2 theta (dwell time 1 s). Background subtraction was performed using DIFFRAC.EVA software V5.1. The surface morphologies of the thin films were studied by an atomic force microscope (AFM) using Park Systems XE 100, with dedicated XEI Software 5.2 Build 1 (Suwon, Republic of Korea), and operated in noncontact mode with silicon AFM probes featuring a tip radius <10 nm.

### OFET Characterization

The characteristics of organic field-effect transistors (OFET) were evaluated using a bottom-gate and bottom-contact geometry with prefabricated high-density platinum OFET chips obtained from Ossila. These chips consisted of 20 devices, each with a constant channel width of 2.5 mm and variable channel lengths ranging from 2 to 10 μm. The prefabricated substrate consists of highly doped p-type silicon with a 300 nm silicon oxide gate dielectric layer grown on both sides, serving as insulation. The source and drain contacts were fabricated with a 5 nm titanium adhesion layer beneath a 100 nm platinum layer. Additionally, a platinum gate contact was deposited along one edge of each substrate. Solutions of P3HT-A, BCP, and SBCP were prepared at a concentration of 5 mg mL^–1^ in toluene. These solutions were gently deposited on the substrate using the drop-casting method at a temperature of 40 °C. The output (*I*_DS_ versus *V*_DS_) and transfer characteristics (*I*_DS_ versus *V*_G_) of the devices, with a channel length of 2 μm, were measured using an OFET high-density board equipped with an Ossila source measure unit system. Field-effect mobility was calculated from the linear region from the standard (eq 1):
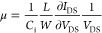
1where *I*_DS_ is drain-source current, μ is the field-effect mobility, *W* and *L* are the channel width and length, *C*_i_ is the capacitance per unit area of the gate insulator (*C*_i_ = 1.09 × 10^–8^ F cm^–2^), and *V*_G_ is the gate voltage.

### Synthesis of Alkyne Functionalized (P3HT-A)

Alkyne functionalized poly(3-hexylthiophene) was prepared following the methods described by Kumari et al. 2015.^17^ 2-Bromo-5-iodo-3-hexylthiophene (0.855 g, 2.6 mmol) was introduced into a three-neck round-bottom flask under argon and then purged under reduced pressure to remove moisture and oxygen. Anhydrous THF (15 mL) was introduced via a syringe, and the solution was stirred and cooled at 0 °C. A 2.3 mL solution of *i*-PrMgCl in THF (1.3 mL, 2.6 mmol) was then added via syringe, and the mixture was stirred at 0 °C for 2 h. The reaction mixture was diluted to 20 mL with THF, followed by the addition of Ni(dppp)Cl_2_ (27 mg, 0.05 mmol). The solution was heated to 35 °C and stirred for 30 min before cooling to 0 °C. Subsequently, 0.5 M ethynylmagnesium bromide (2.6 mL, 1.3 mmol) was added, and the mixture was stirred for an additional 10 min. The reaction was quenched with methanol, resulting in a dark-purple solid, which was filtered and washed with excess methanol. To remove oligomer or low molecular weight fractions, the product was reprecipitated in hexane, filtered, and washed until the filtrate was clear. The resulting polymer was dried under the vacuum. The ^1^H NMR spectrum of the polymer matched the reported data, showing a terminal ethynyl peak at 3.52 ppm, and gel permeation chromatography indicated an Mn ≃ 14 K and a polydispersity index of 1.44. ^1^H NMR (400 MHz, CDCl_3_): δ (ppm) = 0.82-0.97 (m, 3H, CH_3_), 1.28-1.46 (m, 6H, CH_2_), 1.67-1.75 (m, 3H, β-CH_2_), 2.57-2.80 (m, 2H, α-CH_2_), 3.52 (s, 1H, C≡CH), 6.97 (s, 1H, Har)

### Synthesis of P3HT-Block-Poly(ethylene Glycol) (P3HT-b-PEG)

**Scheme 1:**
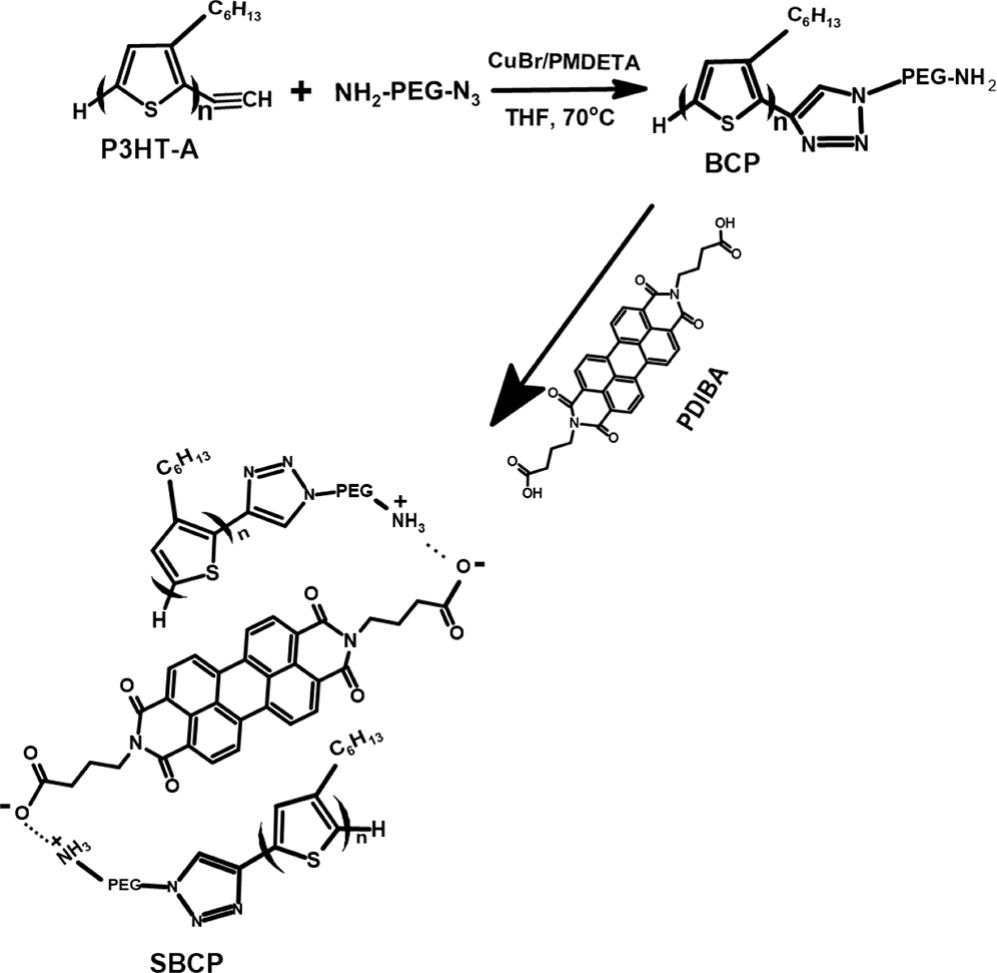
Schematic Representation of Synthesis of Poly(3-hexylthiophene)-Block-Poly(ethylene Glycol) (P3HT-b-PEG) Rod-Coil Block Copolymer (BCP) and Formation of Supramolecular Block Copolymer (SBCP) through Ionic Interaction between P3HT-b-PEG (BCP) and Perylene Diimide Butyric Acid (PDIBA) NH_2_–PEG–N_3_: azide-poly ethylene glycol-amine; P3HT-A: poly(3-hexylthiophene)-alkyne.

The synthesis of P3HT-b-PEG was conducted as follows (Scheme 1).^32^ A 50 mL round-bottom flask equipped with a three-neck stopcock was flame-dried and allowed to cool to room temperature. Under an argon atmosphere, a mixture of P3HT-A (50 mg, 3.5 μmol), azido-terminated PEG (4.5 mg, 4 μmol), Cu Br (1.1 mg, 8 μmol), PMDETA (1.66 μL, 8 μmol), and THF (30 mL) was introduced into the flask. The mixture was vigorously refluxed and stirred at 70 °C under argon for 2 days. The reaction was quenched by adding an excess of methanol. The crude polymer was then washed successively by Soxhlet extraction with methanol, and the solvent was removed by evaporation, yielding a purple solid. The obtained product was characterized by nuclear magnetic resonance (^1^H NMR), Fourier transform infrared (FT-IR) spectroscopies, and gel permeation chromatography.

### Synthesis of Amino Butyric Acid-Functionalized Perylene Diimide (PDIBA)

The synthesis of PDIBA was carried out using a conventional condensation method as described by Keum et al. 2021.^33^ In brief, a solution containing 2.5 mmol of perylene-3,4,9,10-tetracarboxylic dianhydride, 5.5 mmol of 3-aminobutyric acid, and 10 g of imidazole was prepared and heated under nitrogen at 140 °C for 8 h, followed by cooling at room temperature. The reaction mixture was poured into water and filtered to remove unreacted 3,4,9,10-tetracarboxylic dianhydride. The filtrate solution was then precipitated by adding 1 M HCl, filtered, and washed two or three times with distilled water to remove excess imidazole. The resulting dark red solid PDIBA was dried in a vacuum oven at 80 °C and characterized by ^1^H NMR (400 MHz, DMSO-d6, temp 40 °C, δ in ppm): 8.52 (d, 4H, PDI), 8.25 (d, 4H, PDI), 4.07 (t, 4H, CH_2_–N–Ar), 3.22 (t, 4H, CH_2_–COOH), 1.92 (t, 4H, – CH_2_−), 11.94 (s, 2H, OH).

### Preparation of Supramolecular Block Copolymer (SBCP)

A typical procedure involved dissolving 25 mg (1.6 μmol) of BCP and 7.96 mg (1.6 μmol) of PDIBA in 20 mL of a mixed solvent (THF/DMSO 8/2 v/v) in a glass bottle and sealing it. The mixture was sonicated for 30 min and stirred at 60 °C for 24 h, followed by cooling naturally to ambient temperature without stirring. Th solvent was then removed under vacuum, and 15 mL of chloroform was added. The solution was filtered using a 0.5 μm syringe filter, and the material was dried and stored.^34^ The final product was characterized by FT-IR and ^1^H NMR.

## Results and Discussion

**Figure 1:**
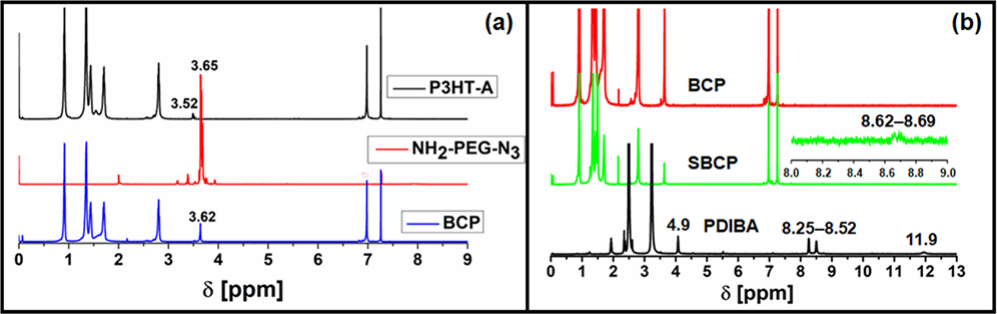
Comparison graph ^1^H NMR spectrum. (a) Poly(3-hexylthiophene)-alkyne (P3HT-A), polyethylene glycol (PEG), and block copolymer (BCP) in CDCl_3_ and (b) BCP, supramolecular block copolymer (SBCP) in CDCl_3_, and perylene diimide butyric acid (PDIBA) in DMSO.

**Figure 2:**
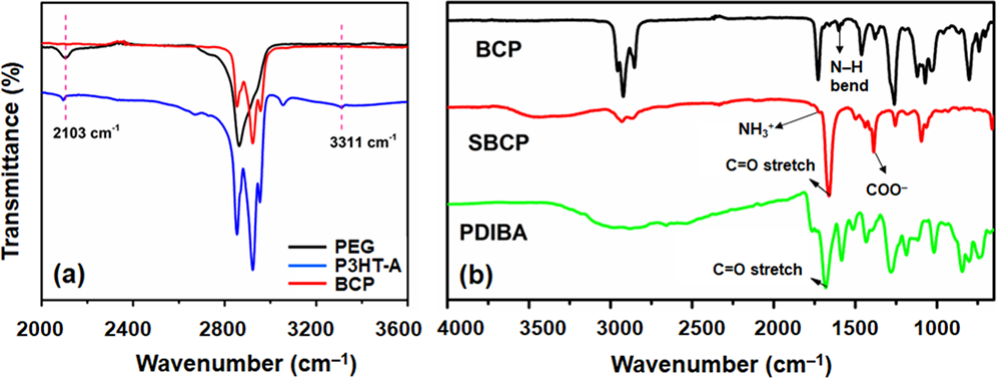
Comparison transmittance spectra obtained from Fourier transform infrared spectroscopy-attenuated total reflectance. (a) Characterize poly(3-hexylthiophene)-alkyne (P3HT-A), polyethylene glycol (PEG), and their block copolymer (BCP). (b) Depicted spectra of BCP, supramolecular block copolymer (SBCP), and carboxyl-functionalized perylene diimide butyric acid (PDIBA).

Scheme 1 illustrates the synthetic pathway for P3HT-b-PEG (BCP), which involves the combination of Grignard metathesis polymerization and click chemistry, followed by the ionic interaction between BCP and butyric acid-functionalized perylene diimide, resulting in SBCP. The successful synthesis of P3HT-A and BCP was confirmed through ^1^H NMR (Figure 1) and FT-IR (Figure 2) analysis. In the NMR spectrum, the terminal alkyne peak of P3HT-A at δ 3.52 ppm is replaced by a PEG proton at δ 3.62 ppm in BCP (Figure 1a). Additionally, FT-IR spectra show the disappearance of the azido stretching frequency at ∼2110 cm^–1^ and the alkyne proton frequency at ∼3311 cm^–1^ and appearance of a strong peak at 802 cm^–1^ for N–H wagging and a very small peak for N–H bend at 1602 cm^–1^ in BCP (Figure 2a).

The number-average molecular weight and the polydispersity index of P3HT-A and BCP were determined by GPC, yielding values of 13500 and 1.40 for P3HT-A and 14 700 and 1.43 for BCP, respectively (Figure S1). Synthesized P3HT-A exhibits a high regioregularity of 96%, as determined by ^1^H NMR spectra. The characteristic methylene proton in the spectrum shows peaks at δ 2.8 ppm, indicative of head-to-tail (HT) arrangements, and a peak at 2.5 ppm corresponds to head-to-head (HH) arrangements. The overall regioregularity was calculated by analyzing the relative integrals of the HT peaks with respect to the total integral of both HT and HH peaks.^35^ Furthermore, the interaction between BCP and PDIBA through ionic interactions was also confirmed by ^1^H NMR and FT-IR. In the FT-IR spectrum, a broad and intense absorption band with a small shoulder is observed, spanning from 1602 to 1712 cm^–1^, primarily due to the −C=O stretching vibration. Additionally, a characteristic peak at 1388 cm^–1^ with a high intensity indicates the formation of COO^–^ through the ionic interaction between BCP and PDIBA, while the small shoulder peak at 1725 cm^–1^ and broadening and overlapping the N–H bending vibration at 1602 cm^–1^ with the C=O peak of PDIBA show the signal of the formation of NH_3_^+^. The CH_2_ stretching bond also broadens, with reduced intensity compared to BCP and higher intensity relative to PDIBA.^36^ Additionally, the presence of an aromatic proton at a higher chemical shift of ∼8.6 ppm in the ^1^H NMR spectrum confirmed the successful synthesis of SBCP (Figure 1b).

### Optical Analysis

**Figure 3:**
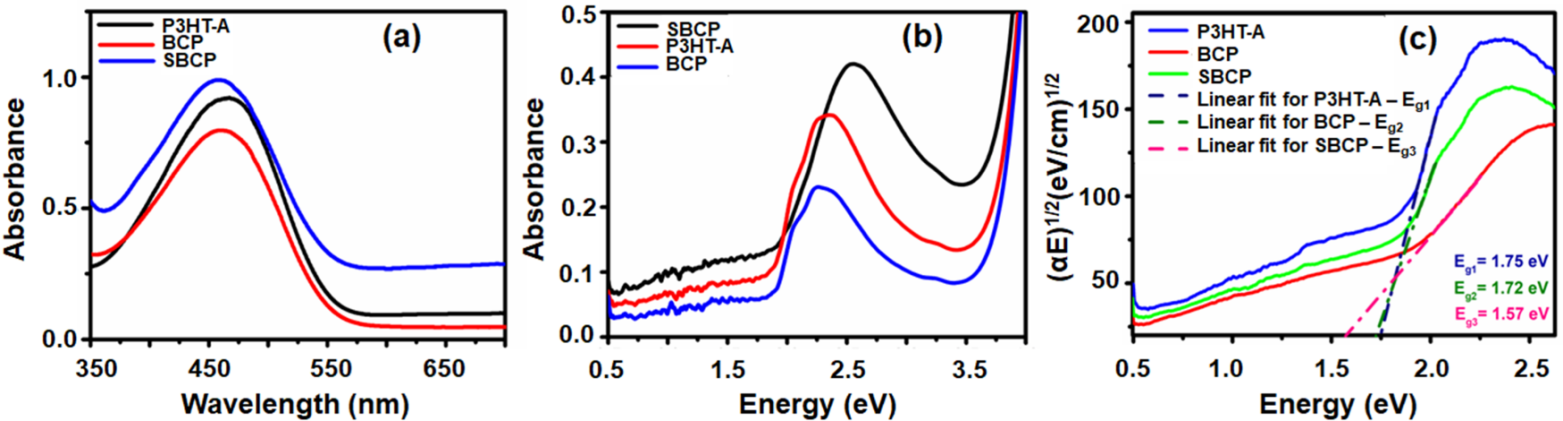
Absorption spectra of poly(3-hexylthiophene)- alkyne (P3HT-A), block copolymer (BCP), and supramolecular block copolymer (SBCP). (a) In solution and (b) in films deposited on glass. (c) Optical energy gaps of P3HT-A, BCP, and SBCP films, determined graphically using the Tauc method.

The UV–vis absorption spectra of P3HT-A, BCP, and SBCP were measured in both dilute toluene solution and thin film (Figure 3a,b). The thin films were deposited by drop casting from a toluene solution (2 mg mL^–1^) onto a glass substrate, followed by an annealing treatment at 160 °C. This annealing step was performed prior to all measurements, with the exception of thermal measurements, to ensure the proper formation and ordering of the films. The annealing process improves the crystallinity and molecular alignment within the films, which in turn can significantly influence their photophysical properties. In the solution absorption spectra, P3HT-A displayed a prominent absorption peak at 465 nm, which was attributed to the π–π* transition of the conjugated system, a characteristic of P3HT-A.^37^ Conversely, the absorption spectrum of BCP exhibited a peak at 462 nm with lower intensity, indicating that slight changes correspond to the P3HT-A block, consistent with previous research findings.^38^

The absorption maxima of SBCP showed a bathochromic shift of approximately 7 nm relative to P3HT, which was attributed to intramolecular interactions between the perylene moiety and the thienyl ring as well as molecular aggregation of the PDIBA crystals (Figure 3a). For the photophysical characterization of the materials in the solid state, we employed transmission spectra to obtain normalized absorption spectra. In our observations, we noted that the absorption maxima of all materials in the solid state exhibited a noticeable red shift compared to their corresponding peaks in solution.

Furthermore, the observed red shift in the absorption spectra can be attributed to enhanced molecular ordering present within the thin films of materials. The improved ordering in the solid state likely leads to increased conjugation and intermolecular interactions, resulting in the observed spectral shift. P3HT-A and BCP reveal almost similar absorption peaks at energy levels of 2.34, 2.19, and 2.02 eV. The presence of the absorption peak at 2.34 eV corresponds to the intrachain π–π* transition within P3HT-A, while the vibrational peaks observed at 2.19 and 2.02 eV can be associated with interchain π–π stacking interactions. However, the more prominent vibronic peak that appeared in BCP may be due to its more ordered structured.^39^ In contrast, the SBCP film displays a noticeable red shift of the absorption peak at an energy level of 2.54 eV and loss of vibronic structure at 2.02 eV compared to P3HT-A and BCP (Figure 3b). This phenomenon is attributed to ionic interactions between BCP and the bulky aromatic PDIBA group, including the perylene cores, which promote H-aggregation behavior and support the self-assembly of PDI derivatives with BCP. The energy band gaps of P3HT-A, BCP, and SBCP were determined and compared using their absorption spectra (Figure 3c). We have used the Tauc relationship,^40^ which is usually employed for amorphous semiconducting materials and was used to calculate the energy gaps (*E*_g_).^41,42^ This involved a linear extrapolation of the plot (α*E*)^1/2^ versus energy *E*. The energy gaps of P3HT-A and BCP are quite similar. However, in the case of SBCP, it has a notable lower value compared to pure P3HT and BCP. This discrepancy is attributed to the intermolecular interactions between the thienyl ring and the perylene core within SBCP.

### Ellipsometric Analysis

Spectroscopic ellipsometry is a convenient reflective optical technique used to measure changes in the polarization parameters (Ψ and Δ) of a light beam. This method is employed to examine the degree of anisotropy and to determine the thickness of the thin film. Ψ represents the amplitude of the polarization ellipse, while Δ denotes the phase shift between the −*p* and −*s* electric vectors of the electromagnetic beam before and after reflection from the sample surface. Ellipsometric angles are related via the main ellipsometry equation:
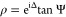
where ρ is the complex reflectance ratio.

**Figure 4:**
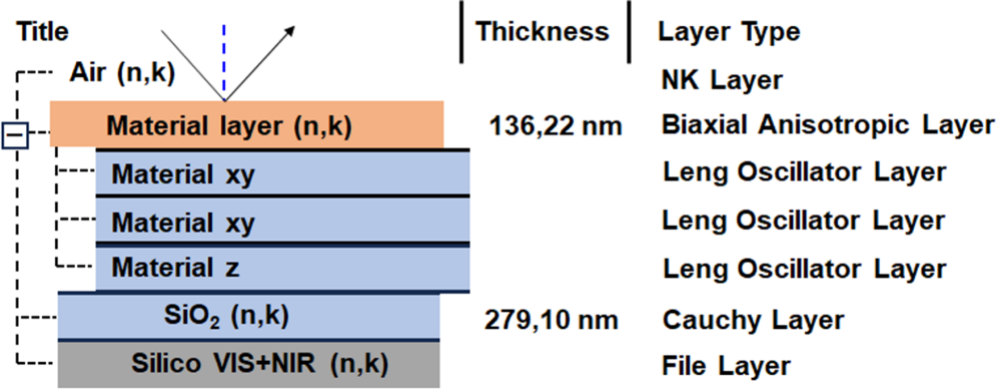
Ellipsometric model was applied for optical analysis.

This ρ coefficient depends on the dielectric functions and is determined theoretically for a specific optical system. This parameter can also be determined theoretically by taking into account the dielectric functions of all optical phases and their thicknesses.^43^ In the complex dielectric functions ε = ε_1_ + *i*ε_2_, ε_1_ is the real and ε_2_ is the imaginary part of dielectric function, and the thickness of the individual films included in optical systems has to be fitted ellipsometrically. In our case, the optical system consists of the four layers(Figure 4).

**Figure 5:**
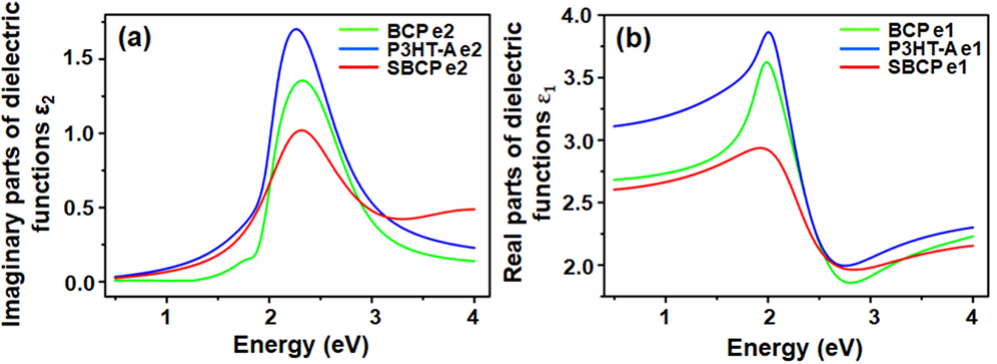
Dielectric functions of poly(3-hexylthiophene)-alkyne (P3HT-A), block copolymer (BCP), and supramolecular block copolymer (SBCP) films deposited onto silicon substrates. (a) Depicted in imaginary part ε_1_ and (b) in real parts ε_2_.

**Figure 6:**
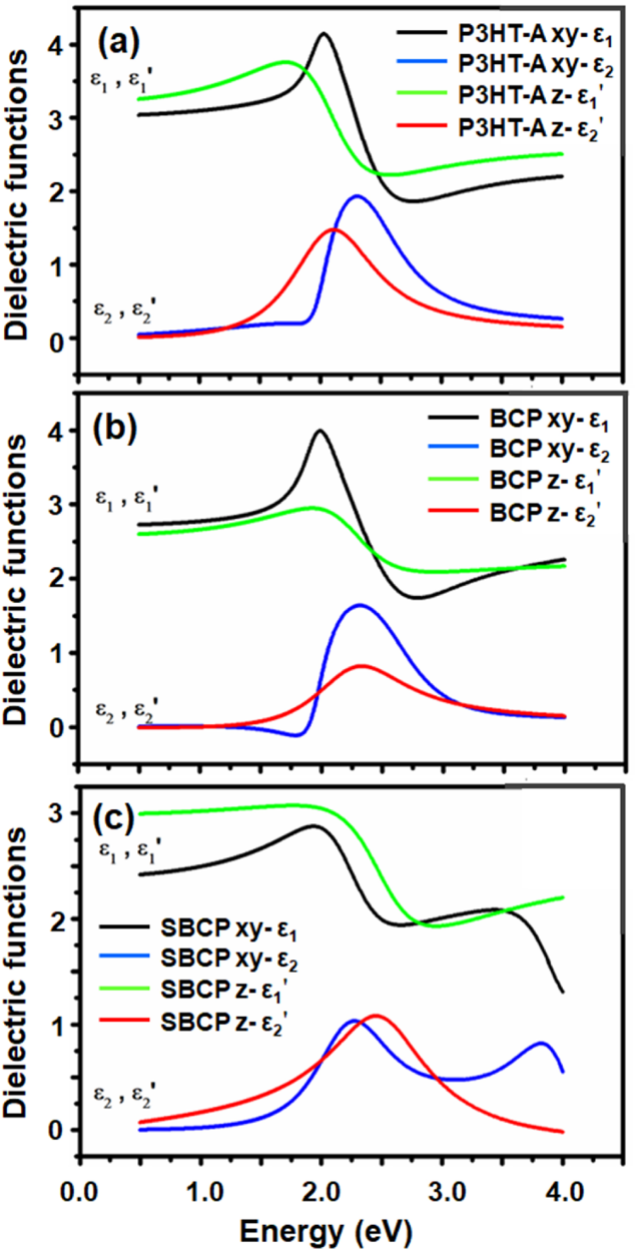
Components of dielectric function ε = ε_1_ + iε_2_ of (a) poly(3-hexylthiophene)-alkyne (P3HT-A), (b) poly(3-hexylthiophene)-block-poly(ethylene glycol) (P3HT-b-PEG), and (c) supramolecular block copolymer (SBCP) films.

The films were deposited onto silicon substrates, which had the same concentration that was used for absorbance measurement, and were covered with ∼300 nm of silicon dioxide. The substrate layers were fitted with file and Cauchy layers.^44,45^ The polymer thin films were fitted using a biaxial anisotropic optical model, and the anisotropy was calculated using three Leng–Lorentz oscillators in the XY plane and one oscillator in the Z direction. The expression for the Leng–Lorentz oscillator layer is defined by eq 2:^46^
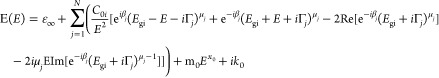
2where the C_0_ is an amplitude, β is the phase, μ is the order of the pole, ω_g_ is the critical frequency point, N is the number of oscillators, and Γ_j_ is a broadening of oscillator. E is the photon energy, where E = *ℏ*ω and *ℏ =* 6.58211 × 10^–16^ eV·s is Dirac’s constant and ω is the frequency of light. The determined dielectric functions are presented in Figures 5 and 6.

The films of P3HT-A, BCP, and SBCP were fitted by using biaxial models, revealing anisotropy in all layers. The values of real ε components and thicknesses of films are presented in Table 1. The values of the dielectric constants for P3HT, BCP, and SBCP are 3.11, 2.68, and 2.60, respectively. The real component value of P3HT-A is consistent with those values reported by Wang et al.^47^ and Knipper et al.^48^ which fall within the range of 3.0–3.6 for ε_1_. The anisotropy was observed in both the XY plane and the Z direction, where the values of ε_1XY_ for P3HT-A and SBCP are lower than values of ε_1Z_ and vice versa for the ε_1_ direction components of BCP. The anisotropic components of dielectric functions are presented in Figure 6. Zhokhavets et al.^49^ reported that the degree of anisotropy in films is strongly dependent on thickness of thin film, with anisotropy increasing as the film thickness decreases which also aligns with our results. Furthermore, the values of refractive indices of P3HT-A, BCP, and SBCP due to present anisotropy are presented in Table 2.

**Table 1:** Thickness and Real Component of Dielectric Functions of Poly(3-hexylthiophene)-alkyne (P3HT-A), Block Copolymer (BCP), and Supramolecular Block Copolymer (SBCP)

	d (nm)	ε_1_ (a.u)	ε_1XY_ (a.u)	ε_1Z_ (a.u)
P3HT-A	102	3.11	3.05	3.27
BCP	135	2.68	2.73	2.61
SBCP	143	2.60	2.42	3.00

**Table 2:** Values of Refractive Indices of Poly(3-hexylthiophene)-alkyne (P3HT-A), Block Copolymer (BCP), and Supramolecular Block Copolymer (SBCP) Films

	n (a.u)	n_*xy*_ (a.u)	n_*z*_ (a.u)
P3HT-A	1.76	1.74	1.80
BCP	1.64	1.55	1.73
SBCP	1.61	1.66	1.60

### Thermal Analysis

**Figure 7:**
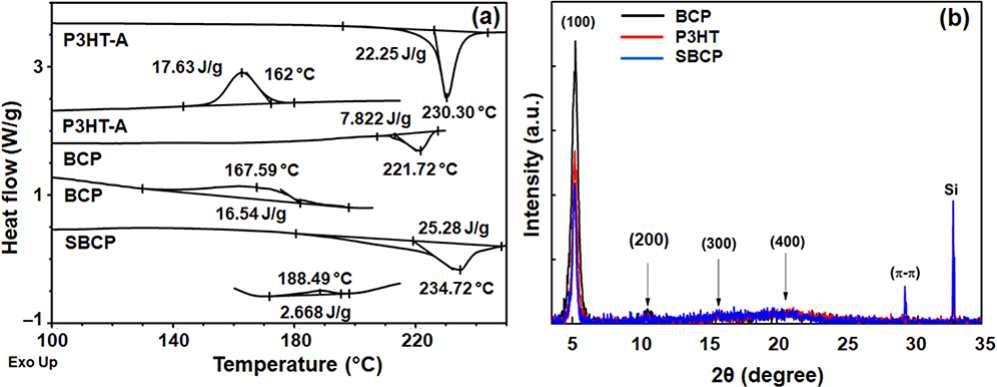
(a) Differential scanning calorimetric and (b) X-ray diffraction spectra of thin films of poly(3-hexylthiophene)-alkyne (P3HT-A), block copolymer (BCP), and supramolecular block copolymer (SBCP).

The thermal properties of P3HT-A, BCP, and SBCP were investigated by using DSC (Figure 7a). Given that all the pure components are semicrystalline, DSC analysis provides valuable insights into the crystallinity and phase behavior of the BCP and SBCP. Table 3 presents the DSC characteristic values for all of the materials, clearly showing the *T*_g_ (glass transition temperature), *T*_m_ (melting temperature), and *T*_c_ (cold crystallization temperature) peaks. These parameters are crucial for understanding the thermal behavior and structural organization of the materials. The DSC curves of the individual PEG-N_3_ and PDIBA are shown in Figure S2. The melting point of pure PDIBA and PEG was found to be 199 and 45.36 °C, respectively, aligning well with previous studies.^32,50,51^ In BCP, the thermal transition of P3HT-A is easily observed, whereas the thermal transition of PEG in the same copolymer is less distinct due to the shorter length of PEG blocks compared to P3HT-A. Thus, this results in the crystallization of P3HT segments dominating over PEG, making it challenging to determine the BCP’s phase separation in the DSC thermogram. With the introduction of PEG, the endothermic peak of P3HT becomes weaker and the exothermic peak becomes broader. Additionally, the melting temperature (*T*_m_) of the synthesized BCP decreases from 230 to 221 °C, indicating that the presence of PEG coils significantly enhances the chain mobility within the BCP. Furthermore, the cold crystallization temperature of BCP slightly increases from 162 to 167 °C, indicating a more ordered arrangement compared to pure P3HT-A. Further analysis shows that the *T*_m_ and *T*_c_ values of SBCP are higher than those of P3HT, due to interactions with the bulky and aromatic groups of PDIBA. These interactions contribute to a higher crystallinity or a more stable ordered state than in the P3HT homopolymer. A comparison of the melt enthalpies of the materials is provided in Table S1.

**Table 3:** Values of the X-ray Diffraction Pattern of Poly(3-hexylthiophene)-alkyne (P3HT-A), Block Copolymer (BCP), and Supramolecular Block Copolymer (SBCP) Films

samples	(100) peak 2θ (degree)	fwhm of (100) peak	crystallite size (A°)	crystallinity (%)	charge carrier mobility (cm^2^/(V s))
P3HT-A	5.17	0.375	235.8	99	2.7 × 10^–3^
BCP	5.20	0.477	185.3	98	0.25 × 10^–3^
SBCP	5.15	0.219	247.4	99	3.82 × 10^–3^

P3HT-A exhibits a Δ*T*m comparable to previously reported values, while the BCP shows lower melt enthalpies compared to pure P3HT-A, due to the incorporation of PEG. However, SBCP demonstrates a higher melt enthalpy, which is attributed to a more ordered and stable crystalline structure within the polymer chains. This enhanced ordering facilitates more efficient packing and stronger intermolecular interactions. A higher melt enthalpy indicates greater crystallinity, as it correlates with the increased energy required to disrupt the crystal structure.

### Surface Morphology

**Figure 8:**
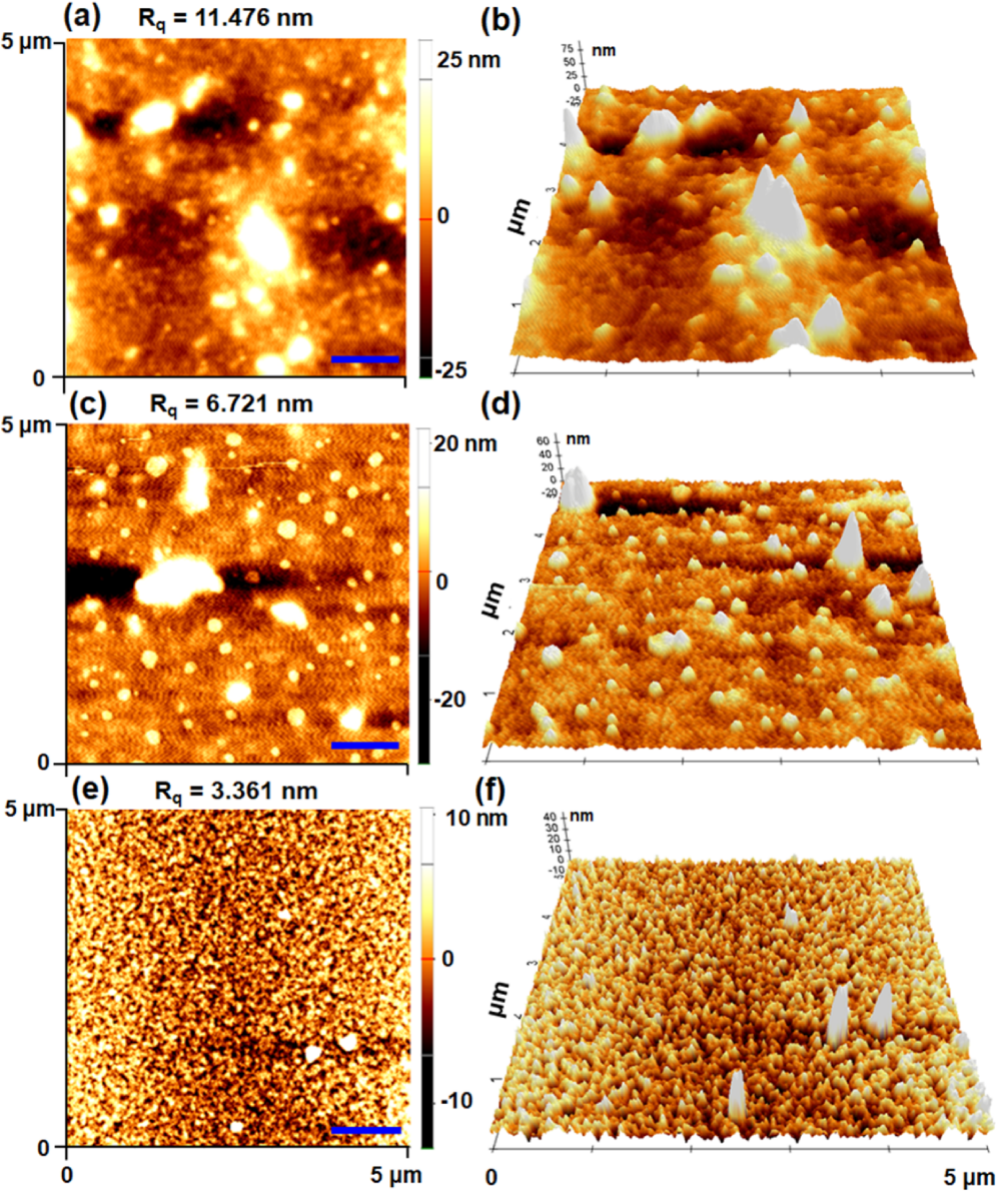
Atomic force micrographs (5 × 5 μm) of thin films of (a, b) poly(3-hexylthiophene)-alkyne (P3HT-A), (c, d) block copolymer (BCP), and (e, f) supramolecular block copolymer (SBCP) prepared by drop casting from toluene (scale Bar = 1000 nm).

To further elucidate, the surface and crystalline structures of the thin films were characterized using X-ray diffraction and AFM analyses (Figures 7b and 8). The AFM topographies (Figure 8) were obtained from silicon wafers that were drop-cast with a 2 mg mL^–1^ toluene solution, examined both in their as-cast and after annealing. The results demonstrated that the as-cast surface structure of P3HT-A exhibited a disordered nanofibrillar structure with larger voids and higher root-mean-square roughness (Rq) value (Figure S3a,b).^52^ However, after annealing, the morphology remained the same, but the surface properties improved due to the crystallization of P3HT on the film’s surface, resulting in a lower Rq value (Figure 8a,b).^53^ We observed that nonannealed films have a higher surface roughness value compared to annealed films due to nonuniform and low film growth rate.^54^ Conversely, the AFM images of the as-cast BCP films showed a flat lamellar structure with low contrast (Figure S3c,d). Upon thermal annealing, the BCP exhibited a phase-separated and more enhanced ordered lamellar nanostructure (Figure 8c,8d).^18^ Furthermore, the AFM images of SBCP revealed a distinct topography compared to pure P3HT-A and BCP. Before annealing, SBCP exhibited a mixed morphology of fibrillar structures with nanodot arrays (Figure S3e,f). However, after thermal annealing, the SBCP self-assembled more compact, uniform grain distribution and densely packed nanofibril crystalline structure, revealing distinct nanophase segregation (Figure 8e,f). The resulted nanostructure is likely due to the combined effects of ionic interactions and strong π–π interactions, which drive PDIBA to organize into ordered arrays at the BCP interface.^18^

To further investigate the effect of PDIBA on the crystallinity and self-assembly of BCP, XRD analysis was performed (Figure 7b). The XRD patterns of the pure polymer P3HT-A and BCP exhibited strong diffraction peaks at 2θ angles ranging from 5.19° to 5.22°, corresponding to a *d*-spacing of 1.69–1.70 nm, along the (100) crystallographic plane. Additionally, broad reflections were observed at 2θ angles of 11.2°, 17.5°, and 22° along second-, third-, and fourth-order (200, 300, and 400) reflections.^55,56^ SBCP also showed the same diffraction peaks, with two additional sharp peaks at 2θ angles of 29.3° and 32.9° where this additional peak is attributed to the π–π stacking distance of the PDIBA core within SBCP and Si (200) reflection, respectively.^57,58^ A sharp and strong (100) peak at 2θ = 5.2° along the Z direction emerges, while for the (010) lattice planes, the peak is not observed, suggesting that all thin films are stacked in an edge-on conformation. The moderate boiling solvent toluene allowing sufficient reorganization time for the polymer to adopt the thermodynamically favored edge-on configuration.^59^ The reflection in the BCP films along 001 is stronger and sharper compared to P3HT-A and SBCP, confirming a higher degree of order within the film, as corroborated by AFM images.^60^ Further analyses using the Diffrac Eva V5.1 program provided insights into the size of crystallites perpendicular to the layer surface (Table 3). Notably, the BCP layer exhibits smaller crystallites and lower crystallinity, measuring 185 Å, while the SBCP layer has larger crystallites at 247 Å and higher crystallinity, compared to the P3HT layer’s crystallite size of 235.8 Å. These XRD findings support the observations from AFM and DSC analysis.

### Field-Effect Transistor Characteristics

**Figure 9:**
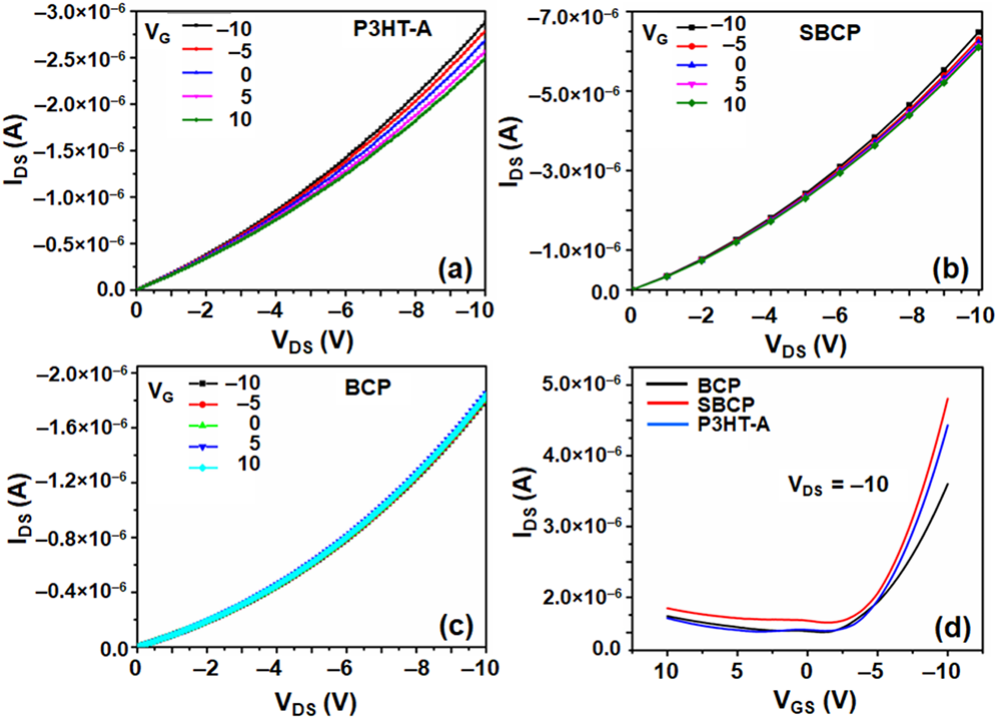
I–V characteristics of poly(3-hexylthiophene)-alkyne (P3HT-A), supramolecular block copolymer (SBCP), and block copolymer (BCP) (a–c) and their output transfer characteristics (d).

Figure 9 shows the drain–source current (I_DS_) at different gate voltages (V_G_) for P3HT-A, SBCP, and BCP (Figure 9a–c), respectively, along with the transfer characteristics (Figure 9d). The electrical characteristics of these devices align well with the previous report.^30^ The fabrication and annealing procedures for our devices followed were consistent with all other measurements conducted to analyze optical and morphological properties, with measurements performed under normal atmospheric conditions. Enhanced output characteristics were observed in the annealed films.^61^ The fabricated OFET devices operated in accumulation mode for the negative gate source voltage and in depletion mode for the positive gate source voltages. Consequently, the I–V characteristics indicate that all devices are configured as p-type. Furthermore, the extracted threshold values of all of the devices were positive.

It has been observed that measured output characteristics do not show distinct gate effects, particularly in BCP and SBCP, though the current changes at lower voltages are consistent with the previous study.^62^ The SBCP device demonstrated a higher drain current and mobility compared to the P3HT-A devices. However, the saturation of OFETs was not achieved at the maximum gate voltage of 10 V. Field-effect mobility was determined by analyzing both output and transfer characteristics at this maximum gate voltage, with mobility calculated from the linear regime of the transfer curve.^63^ Repetition of this experiment yielded consistent results, indicating reproducibility. The results clearly show that while P3HT-A demonstrates slightly higher charge carrier mobility compared to BCP, likely due to the presence of an insulated coil part in BCP, SBCP shows superior drain current and mobility than P3HT-A (Table 3). This enhancement is due to improved morphology and crystallinity properties of SBCP. The morphological and structural analyses suggest that the introduction of PDIBA enhances the crystallinity of the P3HT segments in the films, resulting in the formation of a long-range-ordered nanostructure. A long-range-ordered crystalline structure facilitates better charge carrier transport and higher mobility in conjugated polymers.

## Conclusion

In conclusion, we have successfully synthesized SBCP and investigated the impact of the organic semiconducting molecule PDIBA on the self-assembly behavior of BCP. Ellipsometry analysis confirmed that all thin films exhibited inherent anisotropy. Notably, the synthesized SBCP demonstrated a lower optical band gap compared to pure P3HT-A. The surface topography of P3HT-A revealed a disordered nanofibrillar morphology, while BCP displayed lamellar ordered morphology, as evident in solid state UV–vis spectra and XRD results. However, DSC and XRD analysis confirmed that the presence of insulated PEG part reduced the crystallinity of BCP which can be overcome by introducing an additional ionic interaction between rod-coil BCP, P3HT-b-PEG, with organic semiconducting molecule PDIBA. We found that the SBCP represented both higher crystallite size and crystallinity maintaining an edge-on orientation. Transistor measurements showed that SBCP achieved a higher drain current of 6.67 × 10^–6^ with a higher charge carrier mobility of 3.82 × 10^–3^ compared to pure P3HT-A and BCP. These results obtained from transistor characteristics were reproducible, indicating consistency of the SBCP devices. Overall, this methodology presents a promising strategy for expanding the range of nanostructure morphologies achievable with semiconducting polymers, offering potential advancement in electronic applications.

## Data Availability

Data presented in this article will be available to CMPW-PAN web site.

## Supporting Information Available

Figures S1–S3 and Table S1 are enclosed as a separate document with this manuscript (PDF)
