# Supplementary material for: A Supramolecular Approach to Enhance the Optoelectronic Properties of P3HT-b-PEG Block Copolymer for Organic Field-Effect Transistors
Source: ACS Omega. 2024 Sep 3;9(37):39023–32. doi: 10.1021/acsomega.4c05648 (PMC11411656; doi:10.1021/acsomega.4c05648)
Supplement: Supplementary file 1 — ao4c05648_si_001.pdf [file ao4c05648_si_001.pdf]

Supporting Information

A supramolecular approach to enhance the optoelectronic properties of P3HT-b-PEG block copolymer for organic field effect transistor

Pallavi Kumari[1]*, Barbara Hajduk[1], Paweł Jarka[2], Henryk Bednarski[1], Henryk Janeczek[1], Mieczysław Łapkowski[1,3] Sylwia Waśkiewicz[3]

[1]Centre of Polymer and Carbon Materials, Polish Academy of Sciences, 34 Marie Curie Skłodowska Str., 41–819 Zabrze, Poland

[2]Department of Engineering Materials and Biomaterials, Silesian University of Technology, 18a Konarskiego Str., 41–100 Gliwice, Poland

[3]Department of Physical Chemistry and Technology of Polymers, Faculty of Chemistry, Silesian University of Technology, M. Strzody 9, 44–100 Gliwice, Poland

***Correspondence:** Pallavi Kumari

***E-mail:** pkumari@cmpw-pan.pl

Co-author's E-email:

(B.H.): bhajduk@cmpw-pan.pl

(P.J.): pawel.jarka@polsl.pl

(H.B.): hbednarski@cmpw-pan.pl

(H.J.): hjaneczek@cmpw-pan.pl

(M.Ł.): mieczyslaw.lapkowski@polsl.pl

(S.W.): sylwia.waskiewicz@polsl.pl

Results:

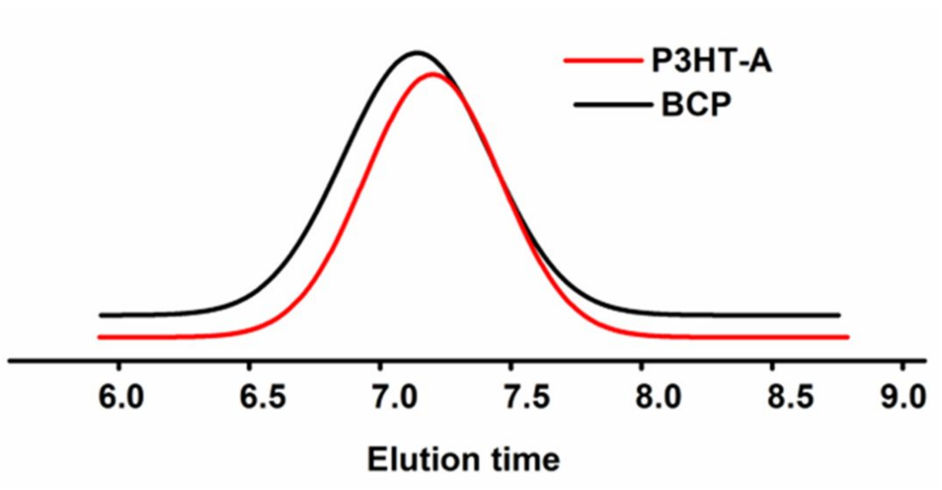

Figure S1

Fig. S1. GPC traces of poly-3-hexyl thiophene (P3HT-A) and block copolymer (BCP).

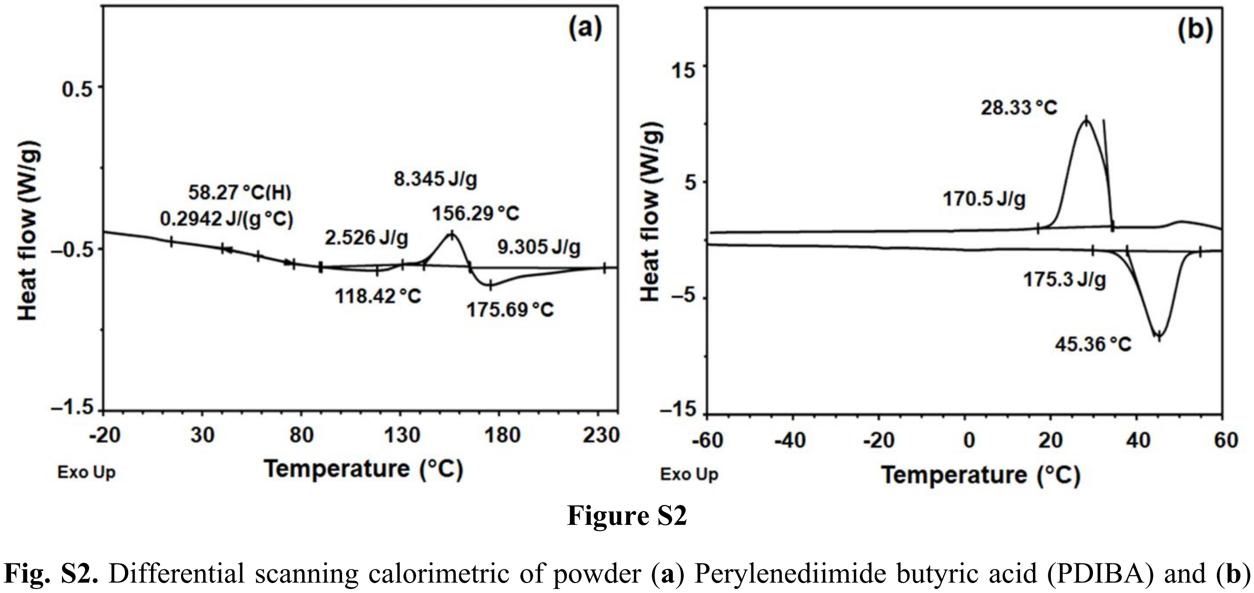

Figure S2

Fig. S2. Differential scanning calorimetric of powder (**a**) Perylenediimide butyric acid (PDIBA) and (**b**) Polyethylene glycol

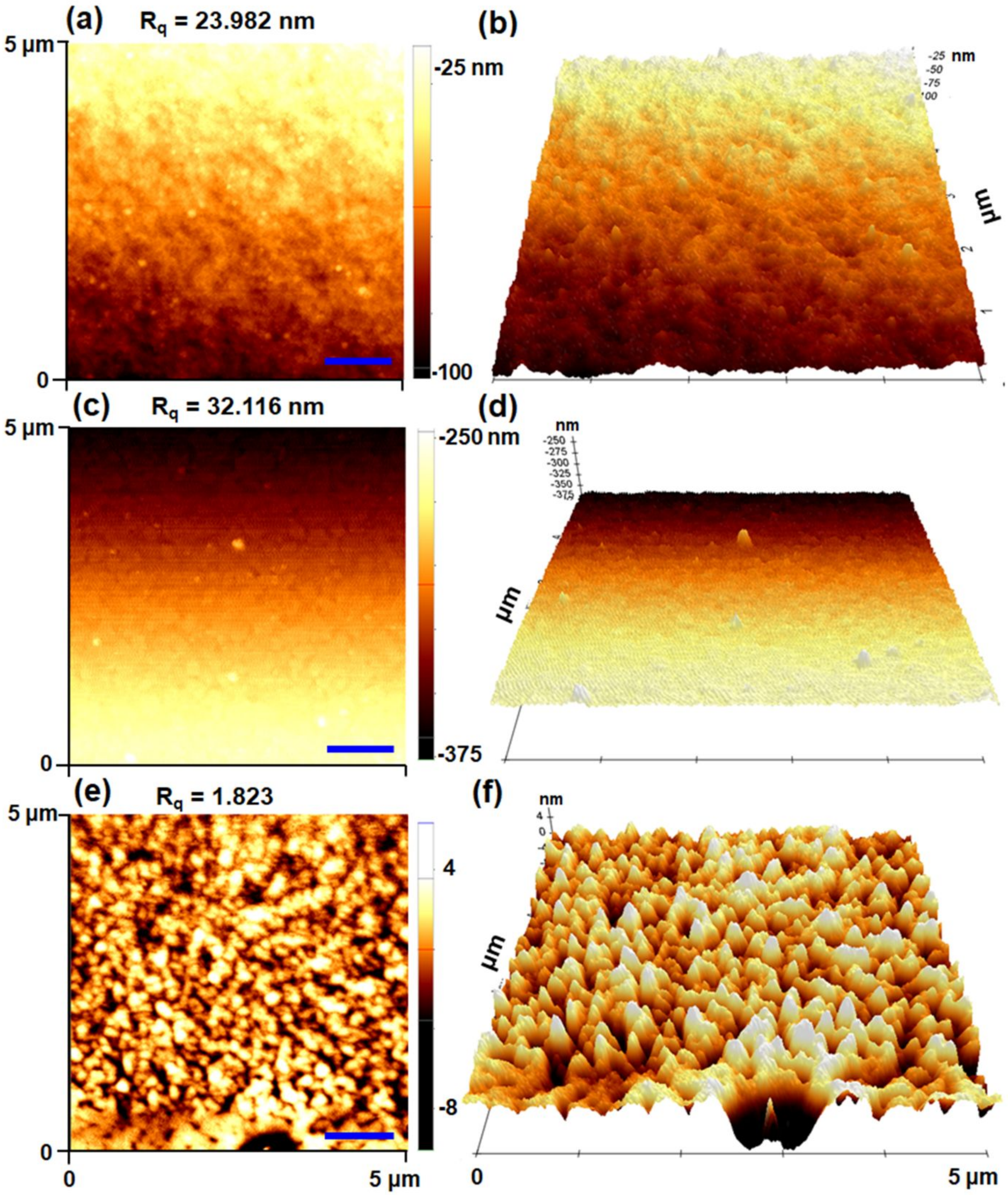

Figure S3

Fig. S3. Atomic force micrographs (5×5 micrometer) of thin films as cast (**a, b**) poly(3-hexylthiophene)-alkyne (P3HT-A), (**c, d**) block copolymers (BCP), and (**e, f**) supramolecular block copolymer (SBCP) prepared by drop casting from toluene (Bar = 1000 nm).

Table S1. Thermal properties of the synthesized poly(3-hexylthiophene)-alkyne (P3HT-A), block copolymers (BCP), and supramolecular block copolymer (SBCP) films prepared by 10 mg mL^{-1} toluene and powder of carboxyl-functionalized perylenediimide (PDIBA) and polyethylene glycol (PEG).

**Sample**	**Tm (°C)**	**Tc (°C)**	**Tg (°C)**	**ΔHm (J/g)**
**P3HT-A**	230	162	20	22.6
**PEG**	54	-	-	193.6
**PDIBA**	199	-	63	10.7
**SBCP**	234	188	106	25.28
**BCP**	221	167	-	7.82
